# Association of D516V, H526Y, and S531L *rpoB* gene polymorphisms and risk factors with rifampicin resistance in *Mycobacterium tuberculosis* isolates from pulmonary TB patients in Northwest Amhara, Ethiopia: cross-sectional study

**DOI:** 10.1186/s12879-025-12351-x

**Published:** 2025-12-18

**Authors:** Kinfe Getachew, Aynias Seid, Nega Berhane

**Affiliations:** 1 Department of Medical Biotechnology, Institute of Biotechnology, University of Gondar, Gondar, Ethiopia; 2 Department of Biology, College of Natural and Computational Science, Debre-Tabor University, Debre-Tabor, Ethiopia

**Keywords:** ARMS-PCR, MDR/RR-TB, Rifampicin resistance, SNP, rpoB

## Abstract

**Background:**

Drug-resistant tuberculosis (DR-TB) continues to pose a threat to public health worldwide. Rifampicin (RIF) resistance is mostly caused by mutations in the *rpoB* gene, which codes for the β -subunit of RNA polymerase and is also an important surrogate marker for multidrug-resistant tuberculosis (MDR).

**Objective:**

This study aimed to detect the *rpoB* gene mutations associated with RIF resistance and identify the risk factors for MDR/ RIF resistance patterns in individuals infected with pulmonary TB.

**Methods:**

A facility-based cross-sectional study was conducted at selected TB treatment center hospitals (Felegehiwot, Debre-tabor, University of Gondar, Debark, and Metema hospitals) from June to December 2023 in the Northwestern Amhara regional state of Ethiopia. A total of 206 pulmonary TB patient’s sputum samples were included. The study participants’ Socio-demographics and clinical and behavioral characteristics were collected through semi-structured questionnaires. Then all GeneXpert^®^ MTB/RI-positive sputum specimens of bacterial isolates were culturedin a conventional egg-based solid Lowenstein-Jensen (LJ) medium. MTB Genomic DNA was extracted using GenoLyze Kit. The allele-specific Amplification Refractory Mutation System Polymerase Chain Reaction (ARMS PCR) approach was employed on whole DNA samples from 206 Culture positive isolates using three distinct codon-specific primers (D516V, H526Y, and S531L).

**Results:**

An isolate is classified as RR-TB if it carries any mutation in the *rpoB* gene. Most Single nucleotide polymorphism (SNP) mutations were observed on *rpoB* S531L 19 (9.2%). Of 206 confirmed clinical isolates, 21 (10.2%) were RIF Resistant, while the remaining 185 (89.8%) were RIF susceptible. Before TB treatment history (AOR = 4.27, CI 1.29–14.20, *p* = 0.02), and Window opening practice of patients (AOR = 6.17, CI 1.22–31.29, *p* = 0.03) were significantly associated with RR-TB development.

**Conclusion:**

The prevalence of RR (RIF Resistant) -TB among TB-confirmed cases was 21 (10.2%). This implies that RR-TB is a serious health problem in the study population. The S531L was the most common mutation conferring resistance to RIF.

**Clinical trial number:**

Not applicable.

**Supplementary Information:**

The online version contains supplementary material available at 10.1186/s12879-025-12351-x.

## Background

Tuberculosis (TB) is a highly contagious infectious disease that is a leading cause of morbidity and mortality worldwide. TB is a bacterial infectious disease caused by *Mycobacterium tuberculosis* complex (MTBC) isolates, usually the human tubercle bacillus, *Mycobacterium tuberculosis* (MTB) [1]. MTB most commonly affects the lungs; despite advances in diagnostic tools, the availability of effective anti-tuberculosis (anti-TB) therapy, and significant global efforts in 2022, 7.5 million people were newly diagnosed with TB and officially reported as TB cases. According to the WHO Global Tuberculosis Report 2024, an estimated 10.8 million people fell ill with TB in 2023 about 134 cases per 100,000 population and 1.25 million deaths were recorded, including 161,000 among people living with HIV. After being temporarily surpassed by COVID-19 during the pandemic, TB has once again become the leading cause of death from a single infectious agent. The report highlights that global TB incidence has rebounded above pre-COVID levels, showing a continued recovery in case detection and reporting compared with 2020 and 2021 [2].

The WHO Global Tuberculosis Report 2024 highlights that in the 30 high TB burden countries, undernutrition, alcohol use disorders, and smoking are among the leading determinants of TB, though their impact varies from country to country. The report further emphasizes that HIV infection remains the most significant contributing factor to TB in many African nations. Collectively, these risk factors along with diabetes play a major role in increasing vulnerability to active TB disease, demonstrating how both biological and social determinants shape the global TB epidemic [2].

The diagnosis and treatment of the disease are more complicated and challenging since the emergence of drug resistance (DR) such as Multidrug resistance (MDR-TB), which is defined as an isolate resistant to at least the two front-line rifampicin (RIF) and isoniazid (INH) drugs. According to the Global TB Report 2022 worldwide in 2022, 73% of people (2.9/4.0 million) diagnosed with bacteriologically confirmed pulmonary TB were tested for rifampicin resistance; among those tested, 5.15% people with MDR/RR-TB and 0.9% people with pre-XDR-TB or extensively DR-TB (XDR-TB) were detected, giving a combined total of 6.1% [2]. DR poses a threat to the viability of successful TB control. MDR-TB hotspots have emerged in areas with ineffective TB control programs and mismanagement of anti-TB drugs, and it has a lower treatment success rate than susceptible TB yet takes more resources to treat [3].

Ethiopia as one of the 30 high TB burden countries and also among the nations with a high burden of TB/HIV co-infection. The country, however, transitioned out of the list of high multidrug-resistant TB (MDR-TB) burden countries in 2021, marking significant progress in TB control efforts [2]. In 2023, Ethiopia’s estimated TB incidence was 146 cases per 100,000 population, with about 8.3% of TB patients co-infected with HIV and a TB mortality rate of 19 per 100,000 among HIV-negative people [4]. In Ethiopia, among nationally reported individuals with MDR/RR-TB, 98% have bacteriologically confirmed pulmonary and/or extrapulmonary TB while 58% have a history of prior TB treatment [1, 5].

Previous studies showed that in MTB strains, unlike other pathogenic bacteria, resistance to anti-TB drugs results from spontaneous gene mutations in specific genes that reduce the bacterium’s sensitivity to antimicrobial agents and affect the effectiveness of anti-TB treatments [6, 7]. RIF resistance mutations are commonly found in the *rpoB* gene, which encodes the β-subunit of RNA polymerase at codons 507–533 in the 81 bp hotspot region of the rifampicin resistance determining region (RRDR) [10, 8]. The mutation frequency can also be variable. *rpoB* mutations are identified in 96.1% of RIF-resistant MTB strains worldwide, and RIF resistance is an important surrogate marker for MDR-TB [9].

The emergence of MDR/RR-TB is considered a significant threat with challenging TB pathogenesis, clinical presentation, and management. Despite extensive studies on DR-TB at global and national levels, specific data regarding RR-TB strains in the Northwestern Amhara region of Ethiopia still need to be included. Most research on DR-TB in Ethiopia addresses broader national or regional trends, leaving a gap in detailed molecular analysis within high-burden areas like Northwest Amhara, Ethiopia. Therefore, this study was performed to detect *rpoB* gene mutations associated with RIF resistance and identify the risk factors of RIF resistance patterns among pulmonary TB patients.

## Materials and methods

### Description of study area

**Fig. 1:**
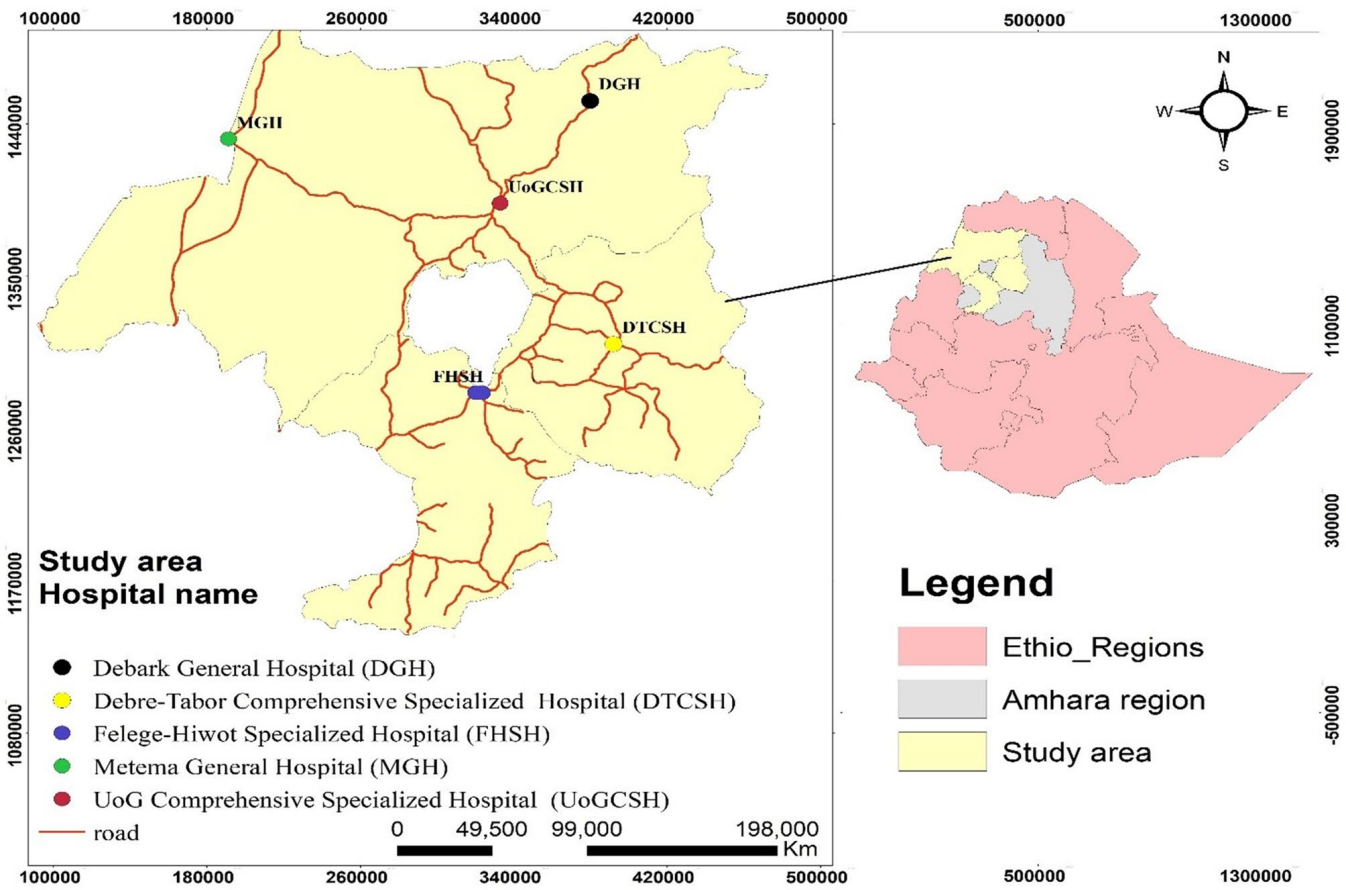
Location map of the study area (ArcGIS)

The study was carried out at selected TB treatment center hospitals in the Amhara regional state in northwestern Ethiopia (Fig. 1). The Amhara Region is located in the northern part of Ethiopia between 8°45’ and 13°45’ North latitude and 36° 20’ and 40° 20’ East longitude. Its land area is estimated at 170,000 square kilometers. Amhara borders the Tigray Region in the North, afar in the East, Oromia in the South, Benishangul-Gumiz in the Southwest, and the country of Sudan in the West. Northwestern part of the Amhara region comprises West Gojjam, South Gondar, Central Gondar, and North Gondar Zones. According to the statistical report of the Central Statistics Agency in 2007, the total population of the Amhara region was 20,018,988 [10].

### Study design and period

A facility-based cross-sectional study was employed on pulmonary TB-suspected individuals from June to December 2023. After collecting and transporting sputum samples Isolation, Identification, and DNA extraction of MTB strains were done at the University of Gondar Comprehensive Specialized Hospital (UoGCSH) TB culture laboratory. The ARMS-PCR assay was done at the University of Gondar, Institute of Biotechnology Molecular Biology Laboratory.

### Source and study population

All GeneXpert^®^ MTB/RIF confirmed pulmonary TB patients were the source population visiting the selected healthcare facilities. Culture-positive samples from confirmed pulmonary TB patients were included as the study population. Culture-based methods ensure that only viable MTB cells are analyzed, which minimizes the potential for false-positive results from non-viable or contaminant DNA in sputum samples.

### Sample size determination and sampling technique

The study participant sample size was determined using a formula for estimating single population proportion with the assumption of a 95% confidence interval and a 5% margin of error [11]. The sample size is calculated by using; $\:n=\frac{{\:z}^{2}\:P\:(1-p)}{d2}$, where; n = Sample size, z = standard normal distribution value at 95% confidence interval (CI), which is 1.96, p = previous prevalence of RIF resistant MTB in Gondar, North Ethiopia, 15.8% [12], d = level of precision or margin of error at 5% (standard value 0.05). n = (1.96)^2^ × 0.158(1-0.158) ÷ (0.05)^2^ = 206. Based on this, 206 pulmonary TB-positive patients among pulmonary TB-suspected individuals were involved in this study. The prevalence (p) of RR-TB was adopted from the study by Jaleta et al. [12]. This specific study was chosen because it was conducted in a regionally similar setting within Ethiopia, focusing on a comparable patient population. Therefore, this prevalence provided the most contextually relevant estimate to ensure an adequate sample size for detecting the association between the targeted risk factors and RR-TB in the current study area.

Five TB treatment hospitals were randomly selected based on the presence of GeneXpert^®^ MTB/RIF TB diagnostic service. The number of study participants to enroll from each selected hospital at the study site was determined proportionally (based on TB patient load during the study period) to meet the sample size. Each culture-positive pulmonary TB patient sample was included consecutively until the target sample size was achieved.

### Inclusion and exclusion criteria

This study included all culture-positive pulmonary tuberculosis (TB) patients who were at least 18 years old and willing to participate in an in-person interview and provide written informed consent. Patients who were unwilling to give interviews or consent were excluded from the study. Critically ill patients who could not offer sociodemographic and clinical information were also excluded.

### Study variables

The detailed personal biographical information on sociodemographic, clinical variables, and behavioral characteristics such as gender, age, religion, residence, marital status, history of contact with active TB cases, window opening status (Habit of patients opening windows in high-risk areas such as rooms, common areas, and spaces where people gather.) whether patients routinely opened windows in their indoor living spaces for ventilation, alcohol consumption, smoking, khat chewing and new/re-treatment TB cases considered as independent variables.

The RIF resistance pattern was dependent on mutations at D516V, H526Y, and S531L.

### Data collection

All the study participant’s profiles (i.e., clinical factors, behavioral, and sociodemographic/ epidemiological information) were collected by trained nurses working at TB treatment centers through a face-to-face interview using a semi-structured questionnaire that had previously been prepared [13].

### GeneXpert MTB/RIF assay and clinical sputum collections

A suspected pulmonary tuberculosis (TB) patient visiting the selected hospitals during the study period was asked to provide deep cough sputum specimens for clinical diagnosis using the GeneXpert^®^ MTB/RIF assay, in accordance with the instructions provided by the World Health Organization and the manufacturer [14]. The GeneXpert^®^ MTB/RIF assay identifies isolates of MTB. For patients confirmed to be positive for pulmonary TB through the GeneXpert^®^ MTB/RIF assay, laboratory professionals collected 5–10 ml of sputum specimens into properly labeled, clean, sterile 50 ml screw-cap disposable plastic Falcon tubes. These specimens were then transported to the TB culture laboratory at the University of Gondar Comprehensive Specialized Hospital (UoGCSH) [15].

### MTB culturing

After sputum collection, all patient sputum specimens were processed using an equal volume of N-acetyl-L-cysteine-NaOH (NALC-NaOH) method for sputum digestion/ homogenization and decontamination with the final 2% sodium hydroxide concentration as described by [16] and then examined using Ziehl-Neelsen (ZN) staining method within 48–72 h of sample collection for identification of AFB of MTB isolates by preparing sputum direct smears with a light microscope [17, 18].

All GeneXpert^®^ MTB/RIF-positive sputum specimens were cultured onto conventional egg-based solid Lowenstein-Jensen (LJ) medium slants containing 0.6% glycerol. The LJ media were prepared and inoculated according to the standard protocol described in the reference [17].

In addition to the AFB positive-smear culture test, the identification of MTB isolates on the LJ medium slant per specimen was confirmed using MPT64 protein detection–based immunochromatographic assay (ICA) (SD BIOLINE TB Ag MPT64 Rapid^®^ commercial test kit, Standard Diagnostics, Inc. South Korea) as per the manufacturer’s guide as previously defined by [19, 20].

### DNA extraction

To enable a fast and effective DNA extraction, the GenoLyze Kit (Hain Life science GmbH, Nehren, Germany) was used according to the manufacturer’s protocol. Briefly, 2 or 3 loops of bacterial culture were transferred into a labeled 1.5 ml screw cap tube. Vortexing resuspended the pellet in 100 µL of Lysis Buffer (A-LYS). The sample was incubated for 5 min at 95 °C in a heat block. After incubation, 100 µL of Neutralization Buffer (A-NB) was added to the lysate, and the sample was vortexed for 5 s. Finally, the mixture was spun down for 5 min at maximum speed (13000 rpm), and the supernatant was transferred into a new tube for storage at -20 °C until further use [21].

### ARMS-PCR assays

For each PCR, 5 µl of genomic DNA was used as a template in a final reaction volume of 20 µl of PCR master mix. This master mix included 0.5 µl of control forward primer, 0.5 µl of ARMS primer, and 0.5 µl of common reverse primer. The PCR was performed using a conventional thermocycler (TECHNE TC-412) with the following program: an initial denaturation at 94 °C for 5 min, followed by 30 cycles of denaturation at 94 °C for 30 s, annealing at 65 °C for 40 s, and extension at 72 °C for 60 s. There was a final extension step at 72 °C for 7 min. Each isolate was subjected to three separate PCRs using three different primer sets. The PCR products were analyzed on a 2% agarose gel after staining with ethidium bromide, and they were visualized using a gel documentation system [22, 23]. The wild-type sequences of strain H37Rv served as a reference and control for the method.

### Primers

The primers (Table 1) in this study were used as described by [22]. The rationale of primer designing for ARMS PCR is that a single nucleotide mismatch at the 3’- OH extremity of the annealed forward primer renders Taq DNA polymerase unable to extend the primer in the PCR under appropriate conditions. Thus, the absence of the specific PCR product, with a positive result for the internal control, reveals a deviation from the wild-type DNA sequence. An additional deliberate mismatch adjacent to the 3′-OH terminuses of the ARMS primer was introduced to enhance discrimination between normal and mutant alleles [22]. A control forward primer was utilized to efficiently anneal to all alleles, paired with a common reverse primer to produce a longer PCR product, serving as an internal control.

**Table 1:** ARMS primers [22]

*rpoB* gene Primers	Sequence 5′-3′	PCR product size in base pairs (bp)
Control Forward Primer	CGAATATCTGGTCCGCTTGC	537
Common Reverse Primer	GTCGACCACCTTGCGGTACG	
ARMS-516 primer	CAGCTGAGCCAATTCA**C**GGA	261
ARMS-526 primer	CGCTGTCGGGGTTG**T**CCC	230
ARMS-531 primer	ACCCACAAGCGCCGAC**A**GTC	216

### Statistical data analysis

All recorded data were entered using EpiData 3.1 data entry software. After the data were cleaned and validated, they were transferred to the Statistical Package for the Social Sciences (SPSS) version 25.0 software (IBM SPSS, Chicago, IL, United States). This study utilized bivariate and multivariate logistic regression analyses to evaluate the associations between risk factors associated with drug resistance. Variables with a p-value of less than 0.25 in the bivariate analysis were further examined through multivariable logistic regression analysis to identify predictors of the outcomes, specifically drug resistance patterns. A p-value of less than or equal to 0.05 (*p* ≤ 0.05) was considered statistically significant.

## Results

### Sociodemographic and clinical characteristics of study participants

A total of 206 sputum samples were collected from new and retreatment cases attending selected TB treatment hospitals from June to December 2023. Among these participants, 128 (62.1%) were males, and 78 (37.9%) were females. The majority of the study participants (105) were in the age group of 18–34 years. The majority of the study participants were new TB cases, 179 (86.9%), TB without/unknown HIV-1 co-infected cases 165 (80.1%), and urban residences, 105 (51.0%) (Table 2).

**Table 2:** Sociodemographic and clinical characteristics of study participants (*N* = 206)

Variables		Categories	Frequency (*N*)	Percentage (%)
Gender of participants		Male	128	62.1
		Female	78	37.9
Age group of patients		18–34	105	51.0
		35–44	43	20.9
		45–64	47	22.8
		> 64	11	5.3
Residency of participants		Rural	101	49.0
		Urban	105	51.0
Marital status of participants		Married	112	54.4
		Single	74	35.9
		Divorced	15	7.3
		Widowed	5	2.4
Number of persons per house		only 1	29	14.1
		2_3	55	26.7
		> 4	122	59.2
Level of education for patients		Illiterate	82	39.8
		1_8	35	17.0
		9_12	56	27.2
		College/university	9	4.4
		Above Diploma	24	11.7
Occupational status of participants		Self-employed	57	27.7
		Governmental	33	16.0
		Farmer	44	21.4
		Housewife	41	19.9
		Student	31	15.0
Prior TB treatment history of patients		New	179	86.9
		Pre-treated	27	13.1
TB/HIV-1 co-infection status of participants		TB with HIV-1 co-infected case	41	19.9
		TB without/unknown HIV-1 co-infected case	165	80.1
Window opening practice of patients		Yes	74	35.9
		No	132	64.1
History of previous contact with TB cases		Yes	54	26.2
		No	139	67.5
		Unknown	13	6.3
Alcoholic status of participants		Alcoholic	48	23.3
		Non-alcoholic	158	76.7
Khat chewing habit of participants		Yes	14	6.8
		No	192	93.2
Smoking status of participants		Smoker	16	7.8
		Non-smoker	190	92.2

### Detection of *rpoB* mutation by ARMS PCR

The ARMS primers employed were complementary to the Corresponding sequence of the wild-type gene except for one additional deliberate mismatch at the fourth nucleotide from the 3′-OH terminuses of the primer. This would create two mismatched nucleotides at the 3′ end between the ARMS primer and the mutated codon. A single mismatch at the fourth nucleotide from the 3’ end of the ARMS primer would have little influence on the yield of PCR products. In contrast, the mismatch at the 3′-OH extremity of the primer is obstinate to extension by the Taq DNA polymerase so that amplification from the mutant allele does not occur [22].

**Fig. 2:**
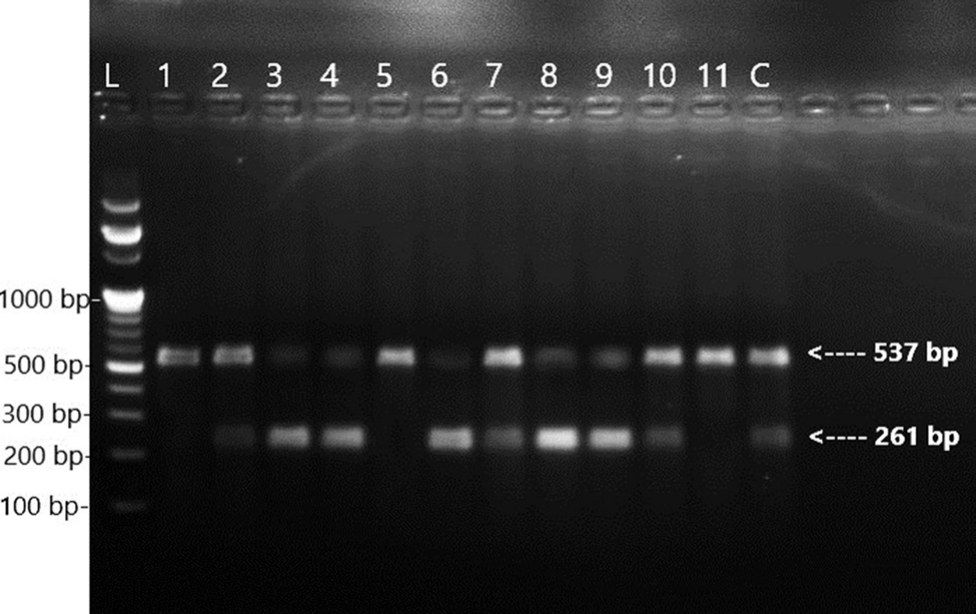
Amplicon patterns of 516

Lane L DNA Ladder 100 bp, Lane 2, 3, 4, 6, 7, 8, 9 and 10 amplifications of *rpoB* 516 wild-type allele specific product 261 bp and internal control 537 bp; Lane 1,5 and 11 absences of amplification of *rpoB* 516 wild-type allele specific product due to mutation indicates RR-MTB isolate, Lane C strain H37Rv wild-type reference allele specific and internal control were amplified (Fig. 2).

**Fig. 3:**
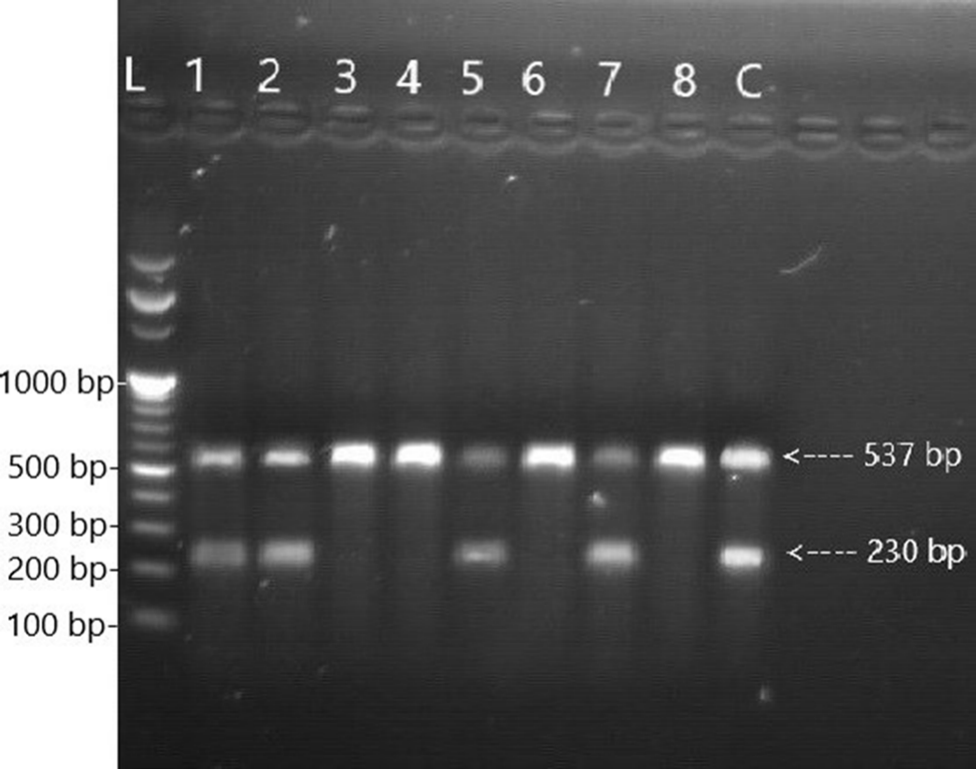
Amplicon patterns of 526

Lane L DNA Ladder 100 bp, Lane 1,2,5 and 7 amplifications of *rpoB* 526 wild-type allele specific product 230 bp and internal control 537 bp; Lane 3,4,6 and 8 absences of amplification of *rpoB* 526 wild-type allele specific product due to mutation indicates RR-MTB isolate, Lane C strain H37Rv wild-type reference allele specific and internal control were amplified (Fig. 3).

**Fig. 4:**
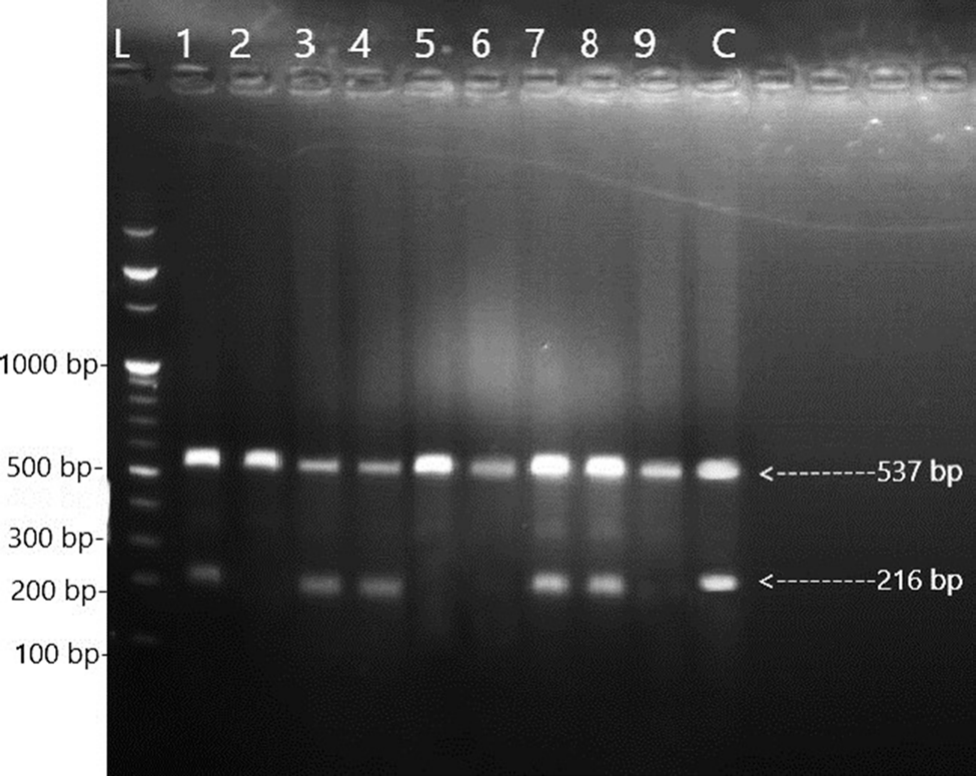
Amplicon patterns of 531

Lane L DNA Ladder 100 bp, Lane 1,3,4,7 and 8 amplifications of *rpoB* 531 wild-type allele specific product 216 bp and internal control 537 bp; Lane 2,5,6 and 9 absences of amplification of *rpoB* 531 wild-type allele specific product due to mutation indicates RR-MTB isolate., Lane C strain H37Rv wild-type reference, allele specific and internal control were amplified (Fig. 4).

### Distribution of *rpoB* mutations

Among the 206 MTB isolates analyzed, 21 (10.2%) were confirmed to be RIF resistant. Each isolate was counted only once when determining the total number of RIF-resistant cases. An isolate is classified as RR-TB if it carries any mutation in the *rpoB* gene at codons 516, 526, or 531. Because several isolates carried mutations at more than one codon, the total counts for individual mutations (S531L, D516V, H526Y, and dual-codon mutations) add up to more than 21. These overlapping mutations reflect multiple changes occurring within the same isolate, not additional RIF-resistant cases. Overall, S531L was the most common mutation (19 isolates, 9.2%), followed by D516V (8 isolates, 3.9%) and H526Y (7 isolates, 3.4%). Some isolates exhibited dual mutations, including 516/531 (7 isolates, 3.4%) and 526/531 (6 isolates, 2.9%).

**Fig. 5:**
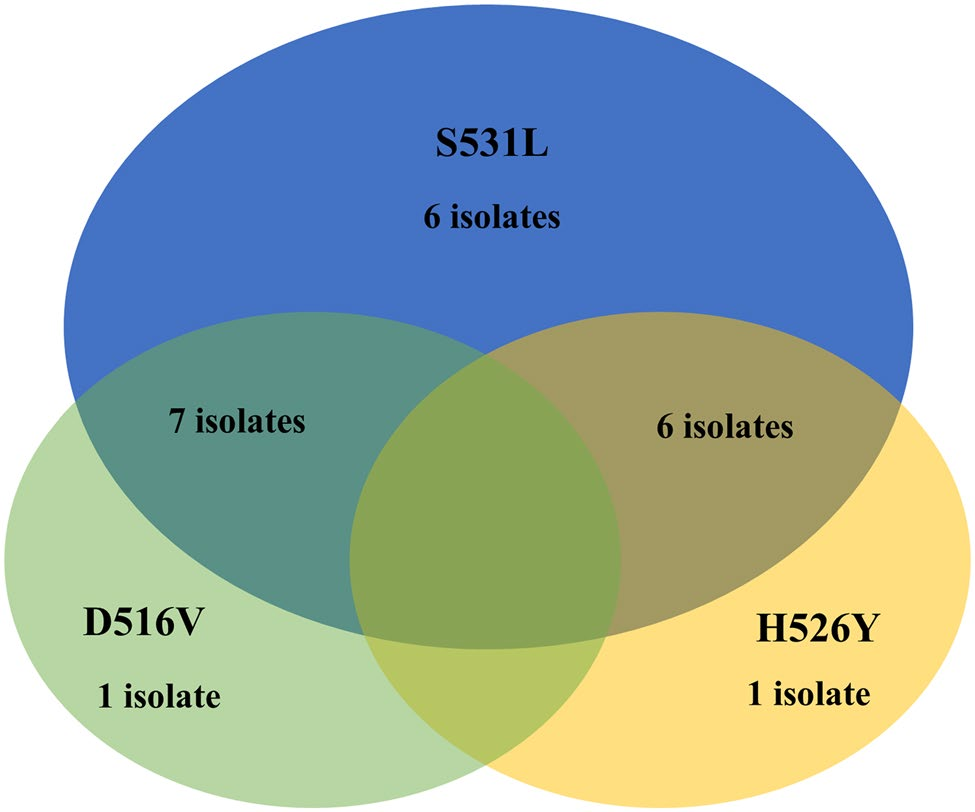
The distribution and overlap of *rpoB* gene mutations (D516V, H526Y, S531L) among RIF resistant MTB isolates (*n* = 21)

The S531L mutation was the most frequent, with 6 single mutations (only at S531L) and notable overlaps occurring between S531L and D516V (7 isolates) and between S531L and H526Y (6 isolates). No isolates carried simultaneous mutations at all three codons (Fig. 5).

### Rifampicin resistance patterns of clinical isolates among study participants

Of 206 confirmed pulmonary TB clinical isolates, 21 (10.2%) were RIF resistant, and 185 (89.8%) were RIF susceptible. Of RIF resistant, 16 (76.2%) were male participants, 13 (61.9%) were among urban residents, 11 (52.4%) were TB with HIV-1 co-infected cases, 11 (52.4%) were newly diagnosed patients, and 10 (47.6%) were previously treated pulmonary TB, Most of RIF resistant were between age 18–34 10 (47.6%) and Married 13(61.9) (Table 3).

**Table 3:** Rifampicin resistance patterns of clinical isolates among study participants (*N* = 206)

Variables	Categories	RR- MTB clinical isolates		Total (N %)
		Resistant (N, %)	Susceptible (N, %)	
Gender	Male	16 (76.2)	112 (60.5)	128 (62.1)
	Female	5 (23.8)	73 (39.5)	78 (37.9)
Age group of patients	18–34	10 (47.6)	95 (51.4)	105 (51.0)
	35–44	3 (14.3)	40 (21.6)	43 (20.9)
	45–64	8 (38.1)	39 (21.1)	47 (22.8)
	> 64	0 (0.0)	11 (5.9)	11 (5.3)
Number of persons per house	only 1	2 (9.5)	27 (14.6)	29 (14.1)
	2_3	2 (9.5)	53 (28.6)	55 (26.7)
	> 4	17 (81.0)	105 (56.8)	122 (59.2)
Marital status of participants	Married	13 (61.9)	99 (53.5)	112 (54.4)
	Single	7 (33.3)	67 (36.2)	74 (35.9)
	Divorced	0 (0.0)	15(8.1)	15 (7.3)
	Widowed	1 (4.8)	4 (2.2)	5 (2.4)
Residency of participants	Rural	8 (38.1)	93 (50.3)	101 (49.0)
	Urban	13 (61.9)	92 (49.7)	105(51.0)
Level of education for patients	Illiterate	10 (47.6)	72 (38.9)	82(39.8)
	1_8	6 (28.6)	29 (15.7)	35 (17.0)
	9_12	4 (19.0)	52 (28.1)	56 (27.2)
	College/university	1 (4.8)	8 (4.3)	9 (4.4)
	Above Diploma	0 (0.0)	24 (13.0)	24 (11.7)
Occupational status of participants	Self-employed	6 (28.6)	51 (27.6)	57 (27.7)
	Governmental	2 (9.5)	31 (16.8)	33 (16.0)
	Farmer	8(38.1)	36 (19.5)	44 (21.4)
	Housewife	4 (19.0)	37 (20.0)	41(19.9)
	Student	1 (4.8)	30 (16.2)	31(15.0)
TB/HIV-1 co-infection status of participants	TB with HIV-1 co-infected case	11 (52.4)	30 (16.2)	41 (19.9)
	TB without/unknown HIV-1 co-infected case	10 (47.6)	155 (83.8)	165 (80.1)
Prior TB treatment history of patients	New	11 (52.4)	168 (90.8)	179 (86.9)
	Pre-treated	10 (47.6)	17 (9.2)	27 (13.1)
History of previous contact with TB cases	Yes	8 (38.1)	46 (24.9)	54 (26.2)
	No	12 (57.1)	127 (68.6)	139 (67.5)
	Unknown	1 (4.8)	12 (6.5)	13 (6.3)
Window opening practice of patients	Yes	2 (9.5)	72 (38.9)	74 (35.9)
	No	19(90.5)	113 (61.1)	132 (64.1)
Khat chewing habit of participants	Yes	3 (14.3)	11 (5.9)	14 (6.8)
	No	18 (85.7)	174 (94.1)	192 (93.2)
Smoking status of participants	Smoker	6 (28.6)	10 (5.4)	16 (7.8)
	Non-smoker	15 (71.4)	175 (94.6)	190 (92.2)
Alcoholic status of participants	Alcoholic	11 (52.4)	37 (20.0)	48 (23.3)
	Non-alcoholic	10 (47.6)	148 (80.0)	158 (76.7)

### Factors associated with RR-TB

The association between potential exposure variables and RR-TB was analysed (Table 4). In bivariate analysis, variables like TB/HIV-1 co-infection status, prior TB treatment history, window opening practice, khat chewing habit, smoking status, and alcoholic status of participants have statistically significant associations with RR-TB.

All variables that were p-value < 0.25 in bivariate analysis considered important and entered into Multivariate logistic regression model and analysis showed that RR-TB was significantly associated with TB/HIV-1 co-infection status of participants (AOR = 3.41, CI 1.12–10.33, *p* = 0.03), previous TB treatment (AOR = 4.27, CI 1.29–14.20, *p* = 0.02) and window opening practice of patients (AOR = 6.17, CI 1.22–31.29, *p* = 0.03).

**Table 4:** Bivariate and multivariate analysis of factors associated with RR-TB

Variables	Categories	RR- MTB clinical isolates		COR (95% CI)	P -Value	AOR (95% CI)	P-Value
		Resistant (N, %)	Susceptible (N, %)				
Gender	Male	16 (76.2)	112 (60.5)	0.48(0.17–1.37)	0.17	1.25 (0.35–4.31)	0.75
	Female	5 (23.8)	73 (39.5)	1.00		1.00	
Age group of patients	18–34	10 (47.6)	95 (51.4)	1.00	0.62		
	35–44	3 (14.3)	40 (21.6)	1.40(0.37–5.37)			
	45–64	8 (38.1)	39 (21.1)	0.51(0.19–1.40)	0.19		
	> 64	0 (0.0)	11 (5.9)	---	---		
Number of persons per house	only 1	2 (9.5)	27 (14.6)	2.19(0.48–10.04)	0.32		
	2_3	2 (9.5)	53 (28.6)	4.29(0.96–19.27)	0.06		
	> 4	17 (81.0)	105 (56.8)	1.00			
Marital status of participants	Married	13 (61.9)	99 (53.5)	1.9(2.00-18.36)	0.58		
	Single	7 (33.3)	67 (36.2)	2.39(0.23–24.48)	0.46		
	Divorced	0 (0.0)	15(8.1)	---	---		
	Widowed	1 (4.8)	4 (2.2)	1.00			
Residency of participants	Rural	8 (38.1)	93 (50.3)	0.61(0.24–1.54)	0.29		
	Urban	13 (61.9)	92 (49.7)	1.00			
Level of education for patients	illiterate	10 (47.6)	72 (38.9)	1.00			
	1_8	6 (28.6)	29 (15.7)	0.67(0.22–2.02)	0.48		
	9_12	4 (19.0)	52 (28.1)	1.81(0.54–6.07)	0.34		
	College/university	1 (4.8)	8 (4.3)	1.11(0.13–9.84)	0.93		
	Above Diploma	0 (0.0)	24 (13.0)	---	---		
Occupational status of participants	Self-employed	6 (28.6)	51 (27.6)	0.28(0.03–2.47)	0.25		
	Governmental	2 (9.5)	31 (16.8)	0.52(0.04-6.00)	0.60		
	Farmer	8(38.1)	36 (19.5)	0.15(0.02–1.27)	0.08		
	Housewife	4 (19.0)	37 (20.0)	0.31(0.03–2.91)	0.30		
	Student	1 (4.8)	30 (16.2)	1.00			
TB/HIV-1 co-infection status of participants	TB with HIV-1 co-infected case	11 (52.4)	30 (16.2)	0.18(0.07–0.45)	0.00	3.41 (1.12–10.33)	0.03*
	TB without/unknown HIV-1 co-infected case	10 (47.6)	155 (83.8)	1.00		1.00	
Prior TB treatment history of patients	New	11 (52.4)	168 (90.8)	1.00	0.00	1.00	0.02*
	Pre-treated	10 (47.6)	17 (9.2)	0.11(0.04–0.30)		4.27 (1.29–14.20)	
History of previous contact with TB cases	Yes	8 (38.1)	46 (24.9)	0.48(0.06–4.21)	0.51	1.10 (0.10-12.65)	0.94
	No	12 (57.1)	127 (68.6)	0.88(0.11–7.38)	0.91	1.34 (0.14–13.05)	
	Unknown	1 (4.8)	12 (6.5)	1.00		1.00	
Window opening practice of patients	Yes	2 (9.5)	72 (38.9)	1.00	0.02	1.00	0.03*
	No	19(90.5)	113 (61.1)	0.17(0.04–0.73)		6.17 (1.22–31.29)	
Khat chewing habit of participants	Yes	3 (14.3)	11 (5.9)	0.38(0.10–1.49)	0.16	1.58 (0.20-12.34)	0.66
	No	18 (85.7)	174 (94.1)	1.00		1.00	
Smoking status of participants	Smoker	6 (28.6)	10 (5.4)	0.14(0.05–0.45)	0.01	2.02 (0.44–9.27)	0.37
	Non-smoker	15 (71.4)	175 (94.6)	1.00		1.00	
Alcoholic status of participants	Alcoholic	11 (52.4)	37 (20.0)	0.23(0.09–0.58)	0.00	2.68 (0.86–8.80)	0.11
	Non-alcoholic	10 (47.6)	148 (80.0)	1.00		1.00	

*Statistically significant: COR: crudes odds ratio; AOR: adjusted odds ratio; CI: confidence interval; HIV: human immunodeficiency virus;

## Discussion

The *rpoB* gene codons 516 (Fig. 2), 526 (Fig. 3), and 531 (Fig. 4) were simultaneously detected using the ARMS-PCR technique. To compare the H37Rv wild-type sequences with related codons, where most point mutations have been identified, the 3′ ends of each allele-specific primer were located in this assay and paired with their corresponding bases. The wild-type allele-specific fragment was amplified because no mutation existed at a related codon. No allele-specific PCR product was produced when a mutation occurred at the targeted codons.

The ARMS assay was used to rapidly detect the mutations in the *rpoB* gene associated with the RR-MTB. Although the method has limitations, its sensitivity is roughly equivalent to the line probe assay [22]. Furthermore, compared with other methods, the ARMS assay is more convenient, less expensive, and easier to perform, since it utilizes only commonly available reagents and equipment [24].

The ARMS assay was employed for the detection of D516V, H526Y, and S531L drug-resistant associated mutation, and the highest frequency was observed on 19 (9.2%) S531L, followed by 8 (3.9%) D516V and 7 (3.4%) H526Y. This result agreed with studies conducted in China and Nepal [22, 25]. Various supporting findings have demonstrated that the prevalence of a common mutation at S531L is highly prevalent globally [26–28]. A combination of mutations was also observed as a double mutation associating codons 526 and 531 in 6 (2.9%), 516 and 531 in 7 (3.4%), and no isolates were found with Triple codon mutations.

Many developing countries are extremely concerned about the rapid increase and emergence of antibiotic resistance in the MTB strain. Laboratory research indicates that MTB DR is rising in Ethiopia. Furthermore, several scholars concurred that the development of MTB’s DR is one of the most significant issues with untreated MTB [29].

RR-TB remains a significant concern in the study population and highlights the need for continued surveillance and effective TB control interventions. The prevalence of RR-TB in this study was 10.2%, the same as that of 10.3% from Debre Markos [30] and 10.2% from Egypt [31]. And a little bit higher than compared to the 9.8% by Araya et al. (2020) [32], 9.9% by Arega et al. 2019 [33] from Hospitals in Addis Ababa, 7.42% by Admassu et al. [34] from Jimma, and 5.5% by Diriba et al. (2021) [35] from Gedeo Zone. However, other studies in the country reported higher prevalence rates: Hamusse et al. (2016) [36] from Arsi, and Jaleta et al. (2017) [12] from Gondar (15.8%), and Mesfin et al. (2018) [37] from Addis Ababa (39.4%). Moreover, different parts of neighboring countries reported higher prevalence in Nairobi, Kenya (30%) [38], Mogadishu, Somalia (35%) [39], and Lagos, Nigeria (23.4%) [40]. However, compared to our findings, the magnitude of RR-TB was lower in reports from Gojam (2.59%) [41], Hawassa (1.24%) [42], and Madda Walabu (4.35%) [43]. Furthermore, WHO 2023 reported 5.15% MDR/RR-TB. The higher RR-TB prevalence in this study is attributed to the inclusion of study participants who were confirmed pulmonary TB patients rather than presumptive TB patients.

In the current study, having a previous history of TB treatment was strongly associated with RR-TB (AOR = 4.27, CI 1.29–14.20, *p* = 0.02). Studies show that TB patients previously treated with anti-TB drugs are 4.2 times more likely to develop RR-TB compared to those without prior TB treatment. This strong association may be because RR-TB strains develop due to repeated cycles of treatment-related killing and regrowth. Repeated medication administration to patients makes the mutant strain dominant. This could support the suggestion that DR results mainly from poor treatment adherence [32, 39].

The co-infection of TB-HIV in this study was found to be high at 19.9%. This finding was lower compared with other studies in Ethiopia, with a rate of 33.3% and 41.9% [44, 45]; other studies in Africa have reported much higher rates than our findings in South Africa, 70% [46] and 93.3% [47]. This inconsistency in the data may potentially be attributed to variations stemming from the contrasting levels of HIV burden within the specific geographic location under consideration. Furthermore, it is plausible that the lack of knowledge and understanding surrounding the co-occurrence of TB and HIV among various societal groups could also contribute to the observed discrepancy.

TB/HIV-1 co-infection status of participants was also significantly associated with RR-TB (AOR = 3.41, CI 1.12–10.33, *p* = 0.03). TB patients with TB/HIV-1 co-infection were 3.4 This is consistent with the guidelines set by the World Health Organization (WHO) and aligns with other studies conducted in the country [32, 48–50]. This significant association may be due to people living with HIV progressing more rapidly to TB disease, which is associated with immune system suppression. The chance of acquiring RR-TB directly will increase in locations where DR-TB is prevalent. However, a lack of association between HIV infection and the development of MDR/RR-TB was observed in studies from Debre Markos and Northwest Ethiopia [29, 30].

This study showed that patients lack of window-opening practice was significantly associated with the prevalence of RR-TB (AOR = 6.17, 95% CI: 1.22–31.29, *p* = 0.03). TB patients who did not open windows were over six times more likely to develop TB/RR-TB compared to those who maintained window-opening practices and adequate ventilation. This finding is consistent with a case-control study conducted in eastern Amhara [51]. It is important to note that inadequate ventilation may not directly cause RR-TB. Rather, poor ventilation increases the risk of TB transmission in general; if a patient is exposed to a drug-resistant strain during transmission, the infection may be RIF-resistant. Therefore, the association reflects the effect of ventilation on transmission risk, not on the development of drug resistance. Madda Walabu supported this result in a previous study in Goba Referral [43].

According to our findings, RR-TB prevalence was 16 (76.2), and 5 (23.8), in males and females, respectively, but gender is not significantly associated with RR-TB. This finding agrees with the eastern Amhara and Ethiopia studies [52, 53]. However, in other studies, gender is significantly associated with RR-TB in Ethiopia, with a higher proportion of RR-TB cases found in female patients [33, 54].

Although in this study, the history of previous contact with TB cases, Khat chewing habit, smoking status, and alcoholic status of participants had a significant association with RR-TB independently, still, they have no significance in the multivariate association. This aligns with findings from earlier studies [42, 50, 55].

### Limitation of the study

This study has some limitations. The sample size was relatively small, which may limit the generalizability of the findings. The ARMS-PCR method used may have reduced sensitivity, especially in samples with low bacterial loads. In addition, some participants may not have recalled all the information accurately during data collection, so the data may be affected by recall bias.

## Conclusion

In this study, ARMS-PCR successfully detected the key rpoB mutations. the majority of mutations occurred at codon S531L followed by codons D516V and H526Y. Thus, ARMS-PCR technique can be a reliable, cost-effective alternative approach for detecting RR-TB associated mutations in resource limited settings. The prevalence of RR-TB among TB-confirmed cases was 10.2%. History of previous TB treatment, TB/HIV-1 co-infection, and lack of window opening practice were significantly associated with RR-TB. Therefore, strengthening DR-TB prevention and control strategies by prioritizing TB patient with previous treatment history and TB/HIV-1 co-infected patients helps to reduce the prevalence of DR-TB among TB-confirmed cases in the study area and similar setting. Furthermore, behavioral interventions such as encouraging consistent window opening practice can limit the transmission of DR-TB by improving adequate ventilation.

## Supplementary Information

Below is the link to the electronic supplementary material.

Supplementary Material 1

Supplementary Material 2

Supplementary Material 3

## Data Availability

The datasets generated and analyzed during the current study are available from the corresponding author upon reasonable request. All relevant data supporting the findings of this study are included in the manuscript and its supplementary information files. Any additional materials or raw data required for reproducibility or further investigation can be provided upon request, while adhering to ethical and confidentiality guidelines.

## Abbreviations

DR: Drug Resistance
INH: Isoniazid
LJ: Lowenstein–Jensen
MDR-TB: Multidrug-Resistant Tuberculosis
MOH: Ministry of Health
MTB: Mycobacterium tuberculosis
NALC-NaOH: N-Acetyl-L-Cysteine Sodium Hydroxide
PCR: Polymerase Chain Reaction
RIF: Rifampicin
RRDRs: Rifampicin Resistance-Determining Regions
RR-TB: Rifampicin Resistant Tuberculosis
SNPs: Single Nucleotide Polymorphisms
TB: Tuberculosis
WHO: World Health Organization
XDR: Extensively Drug-Resistant
