# Supplementary figures and images for: Association of D516V, H526Y, and S531L rpoB gene polymorphisms and risk factors with rifampicin resistance in Mycobacterium tuberculosis isolates from pulmonary TB patients in Northwest Amhara, Ethiopia: cross-sectional study
Source: BMC Infect Dis. 2025 Dec 18;26:115. doi: 10.1186/s12879-025-12351-x (PMC12828965; doi:10.1186/s12879-025-12351-x)

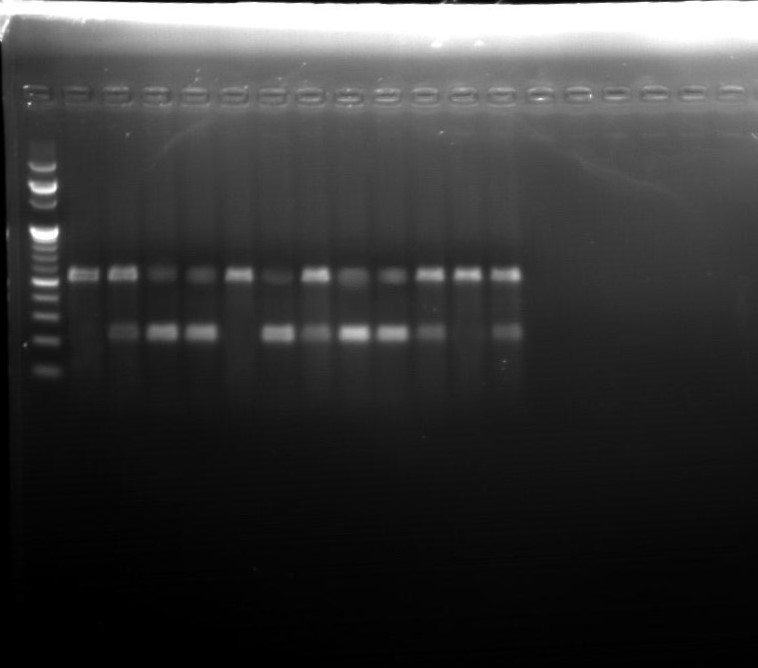

Supplement: Supplementary file 1 — Supplementary Material 1 [file 12879_2025_12351_MOESM1_ESM.jpg]

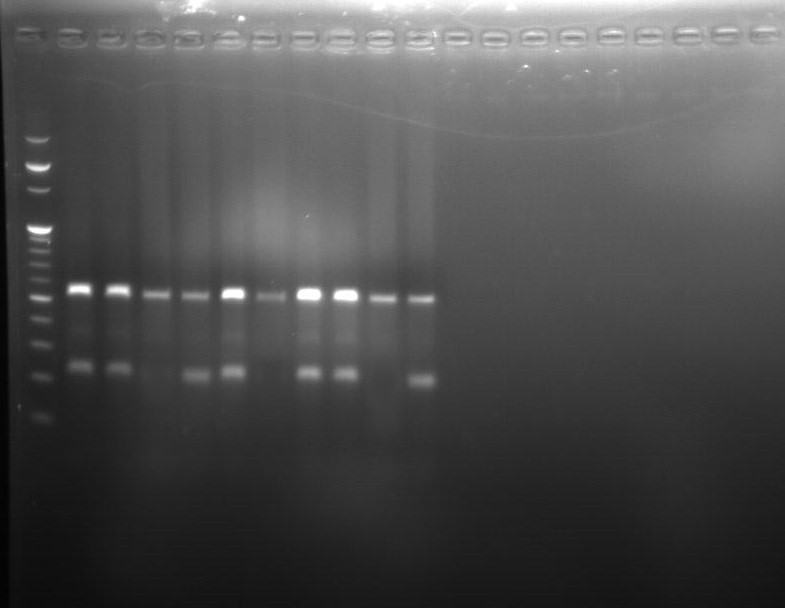

Supplement: Supplementary file 2 — Supplementary Material 2 [file 12879_2025_12351_MOESM2_ESM.jpg]

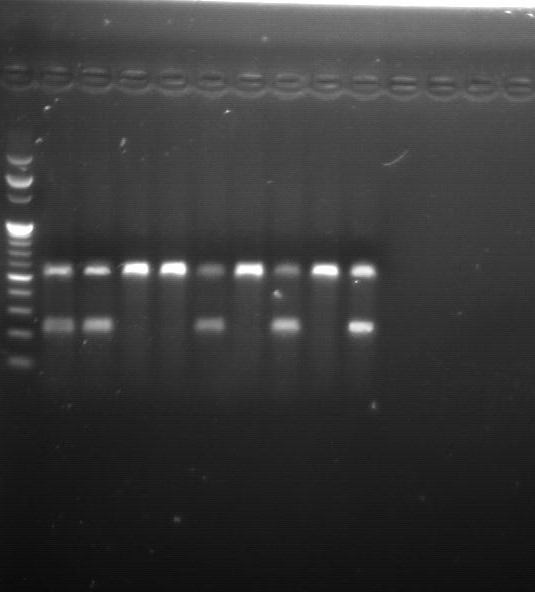

Supplement: Supplementary file 3 — Supplementary Material 3 [file 12879_2025_12351_MOESM3_ESM.jpg]
